# Understanding and using comparative healthcare information; the effect of the amount of information and consumer characteristics and skills

**DOI:** 10.1186/1472-6947-12-101

**Published:** 2012-09-07

**Authors:** Nicolien C Zwijnenberg, Michelle Hendriks, Olga C Damman, Evelien Bloemendal, Sonja Wendel, Judith D de Jong, Jany Rademakers

**Affiliations:** 1 NIVEL, Netherlands Institute for Health Services Research, P.O. Box 1568, Utrecht, 3500 BN, the Netherlands; 2 Department of Public and Occupational Health and the EMGO Institute for Health and Care Research, VU University Medical Center, Amsterdam, The Netherlands

**Keywords:** Healthcare consumers, Comparative healthcare information, Amount of information, Sociodemographic characteristics, Literacy, Numeracy

## Abstract

**Background:**

Consumers are increasingly exposed to comparative healthcare information (information about the quality of different healthcare providers). Partly because of its complexity, the use of this information has been limited. The objective of this study was to examine how the amount of presented information influences the comprehension and use of comparative healthcare information when important consumer characteristics and skills are taken into account.

**Methods:**

In this randomized controlled experiment, comparative information on total hip or knee surgery was used as a test case. An online survey was distributed among 800 members of the NIVEL Insurants Panel and 76 hip- or knee surgery patients. Participants were assigned to one of four subgroups, who were shown 3, 7, 11 or 15 quality aspects of three hospitals. We conducted Kruskall-Wallis tests, Chi-square tests and hierarchical multiple linear regression analyses to examine relationships between the amount of information and consumer characteristics and skills (literacy, numeracy, active choice behaviour) on one hand, and outcome measures related to effectively using information (comprehension, perceived usefulness of information, hospital choice, ease of making a choice) on the other hand.

**Results:**

414 people (47%) participated. Regression analysis showed that the amount of information slightly influenced the comprehension and the perceived usefulness of comparative healthcare information. It did not affect consumers’ hospital choice and ease of making this choice. Consumer characteristics (especially age) and skills (especially literacy) were the most important factors affecting the comprehension of information and the ease of making a hospital choice. For the perceived usefulness of comparative information, active choice behaviour was the most influencing factor.

**Conclusion:**

The effects of the amount of information were not unambiguous. It remains unclear what the ideal amount of quality information to be presented would be. Reducing the amount of information will probably not automatically result in more effective use of comparative healthcare information by consumers. More important, consumer characteristics and skills appeared to be more influential factors contributing to information comprehension and use. Consequently, we would suggest that more emphasis on improving consumers’ skills is needed to enhance the use of comparative healthcare information.

## Background

As a result of the increased emphasis on transparency in quality of healthcare, consumers are more and more exposed to comparative healthcare information, i.e. information about the quality of different healthcare providers [1,2]. They are expected to actively make informed choices for healthcare providers (such as hospitals), treatment options or health plans. The underlying assumption is that by selecting high-quality providers against competitive prices, consumers can contribute to an efficient and patient-centred healthcare system [3,4].

Although consumers are interested in comparative healthcare information [5-9], multiple studies also demonstrated that the use of this information has been limited [3,4] and there is limited evidence of an effect on consumer choice [3,10,11]. Several explanations for this lack of use have been suggested, the most important being the complexity of the information as well as lacking skills of consumers [4,10,12,13].

Comparative healthcare information is typically complex information, consisting of information on a large amount of healthcare providers which can be compared on multiple fairly technical and medical quality aspects (attributes). From the bounded rationality literature it is known that people can process and use only a limited amount - about 4 to 6 aspects - of such multi-attribute information when making choices [14-17]. When too much information is provided, the decision making process may exceed human information-processing skills. People then switch to more heuristic strategies and often base their choice on only a subset of the provided information [18,19], or ignore the information at all. Therefore, researchers in the field of comparative healthcare quality information have called for presenting only a limited amount of the available information [1,2,20].

Concerning consumers’ characteristics and skills, both socio-demographic characteristics [21,22] and more specific variables related to motivation and cognitive capacity to process and use comparative healthcare information are important. Older and lower educated people have more difficulty with the comprehension of comparative information than younger and higher educated people [21,22]. According to Tu and Hargraves [23], persons with a college degree are also more likely to seek healthcare information compared to persons with a high school degree, which will likely influence their motivation to process the information. The same accounts for patients with a chronic illness; they are more likely to seek healthcare information [23].

Numeracy and literacy are two specific skills needed to understand and use comparative healthcare information. Numeracy is defined as the ability to understand and use numbers [24] and literacy is “the ability to understand and employ printed information in daily activities to achieve one’s goals, and to develop one’s knowledge and potential” (OECD 2000, p. X;[25]) Health literacy is a further specification of literacy in the health context, meaning “the degree to which individuals can obtain, process, and understand the basic health information and services they need to make appropriate health decisions.” (Berkman et al. 2011, p 1;[26]. Results of the International Adult Literacy Survey, which was performed in 20 countries, showed that between one-quarter and three-quarters of adults fail to attain a literacy level (numeracy is a subcomponent of this measure) which is minimally necessary for coping with the demands of modern life and work [25]. In the Netherlands, about 1.5 million people (11% of the population) have limited literacy skills [27,28]. A study of Hibbard and colleagues [29], showed that health literacy and numeracy were positively associated with the comprehension of comparative healthcare information.

Active choice behaviour, which means taking an active role in searching for information and making healthcare choices [30], can be seen as a skill related to motivation to use comparative healthcare information. On the one extreme of a continuum there is the consumerist or ‘in control’ patient, on the other extreme is the passive, dependent patient. The consumerist patient or health consumer actively searches for information (internet, medical specialists, family), involves others in the decision making process and is in charge during this process. The passive, dependent patient or health consumer puts someone else in charge (often the medical professional) to carry out the decision making process [30].

It may seem obvious that both the complexity - in particular the large amount of presented information- as well as several consumer characteristics and skills will influence consumers’ understanding and use of comparative healthcare information. Managing the amount of information as a means to facilitate the use of comparative healthcare information seems more easy to accomplish than increasing consumers’ skills. It remains, however, unclear to what extent the amount of information influences the effective use of information when important consumer characteristics and skills are also taken into account. For this purpose, we examined the effects of the amount of information presented and consumer characteristics and skills on several indicators of effective information use, namely comprehension of information, the choice itself, ease of making a choice and the perceived usefulness of the information. Comparative hospital information about a total hip or knee surgery was used in this study as a test case, because it involves an elective surgery for which people have sufficient time to search for information and make a choice. Also, comparative healthcare information about this surgery has been disclosed for several years now in the Netherlands. The following research questions were addressed:

1) What is the influence of the amount of presented information and consumer characteristics and skills on the comprehension of comparative healthcare information, the choice itself, the ease of making this choice and the perceived usefulness of the information?

2) Does the relationship between the amount of information and the outcome measures still exist when consumer characteristics and skills are taken into account?

## Methods

### Design

We employed a randomized controlled experiment in which four subgroups of participants were each provided with a different amount of comparative healthcare information. In an online survey, the four subgroups were shown 3 (group 1), 7 (group 2), 11 (group 3) or 15 (group 4) quality aspects related to a total knee or hip surgery of three different hospitals, respectively (see Table 1). In each condition, we asked participants to choose a hospital and answer a series of questions about the information provided.

**Table 1:** Aspects of comparative healthcare information shown to each subgroup (translated from Dutch)

	**Hospital A**	**Hospital B**	**Hospital C**	**Subgroup**
Conduct of doctors	★★★	★★☆	★★☆	1, 2, 3, 4
Conduct of nurses	★★☆	★☆☆	★★☆	2, 3,4
Pain control	★★☆	★★☆	★★☆	3,4
Information about new medication	★★★	★★☆	★★☆	4
Information provision before surgery	Yes	No	Yes	2,3,4
Procedures to prevent adverse effects of thrombosis	Yes	Yes	No	1,2,3,4
Registration of complications related to THA/TKA	Yes	No	Yes	3,4
National registration of orthopaedic implants	Yes	Yes	Yes	3,4
Transfusion of homologous blood	Yes	Yes	No	4
Specialist areas of orthopaedist	No	No	No	2,3,4
Number of performed total knee- or hip replacements among adults in a year	314	98	244	1,2,3,4
Number of performed total knee- or hip replacements among children in a year	0	1	0	4
Number of orthopaedists in the hospital	8	2	4	2,3,4
Information provision approach	Written information material and briefings	Written information material	Written information material	3,4
Group-hospital admission	Yes	No	No	4

★★★ better than average.

★★☆ average.

★☆☆ worse than average.

The information provided was derived from patient experience indicators measured by the Consumer Quality Index (CQI: the Dutch standard for measuring patient experiences in healthcare), and indicators about hospital services and clinical performance indicators were derived from hospital registrations.

We showed three types of quality aspects which are all used for actual comparative healthcare information in the Netherlands: patient experiences, hospital services and clinical performance. Information on patients’ experiences was derived from indicators measured by the Consumer Quality Index (CQI: the Dutch standard for measuring patient experiences in healthcare [31]). Information on hospital services and clinical performance was derived from hospital registrations. As people can process and use about 4 to 6 aspects in their decision making process, we have chosen to provide the subgroups with less or more aspects around these numbers, with 15 items as the maximum amount of Dutch quality indicators available.

Ethical approval of the study was not necessary as research by means of surveys that are not taxing and or hazardous for patients (i.e. the once-only completion of a questionnaire containing questions that do not constitute a serious encroachment on the person completing it) is not subject to the Dutch Medical Research Involving Human Subjects Act (WMO). Subjects were free to respond to the questionnaire and they were informed about the aim of the survey.

### Participants and data collection

Participants were recruited in two ways. First, patients who underwent or had to undergo a total hip or knee surgery could enrol themselves in this study by contacting the research institute. We posted calls on websites of patient organisations for orthopaedic patients, on websites of Dutch associations for senior citizens, and on the website of the Dutch Federation of Patients and Consumer Organisations (NPCF). As this study was part of a larger research project, patients could also enrol themselves by reporting their interest for this study in an earlier study in which they participated. In total, 76 patients enrolled themselves in this study. These patients were randomly assigned to one of the four subgroups.

Second, the NIVEL “Insurants Panel” was used to recruit participants. Eight hundred panel members were invited. We used stratified random sampling to assign the panel members to the four groups and create four equal subgroups concerning age, gender and educational level. The Insurants Panel consists of approximately 10,000 insurants of one of the biggest Dutch health insurers. The aim of the panel is to gather information on consumers’ experiences with and expectations of health care in general and their health insurer in particular. Members for the panel were recruited through an announcement in the magazine of the health insurer and by calling and asking them to join the panel. Compliance with privacy regulations was approved by the Dutch Data Protection Authority (nr. 1309664).

Data were collected through an online survey in November and December 2009. Participants viewed the comparative healthcare information on the computer screen and answered the questions while they could still view the information. Also, a short explanation of the different quality aspects was provided. A reminder was sent to the non-responders.

### Outcome measures

Table 2 describes the outcome measures. The questionnaire contained five questions about the comprehension of the information. A sum-score of the number of correct responses on these questions was calculated to test the comprehension of the information. Two questions covered respondent’s hospital choice and the ease of making this choice. As for the hospital choice, hospital A scored highest or equal on all aspects compared to hospital B and C and was therefore considered the ‘correct’ answer. The ease of the hospital choice was rated on a visual analogue scale ranging from −3 (very difficult) to 3 (very easy). The perceived usefulness of the information was measured by seven items; factor analysis showed that these items constituted one scale with high reliability (α = .87). After recoding contra-indicative items, the scores were added up and a higher score represented a higher perceived usefulness of the information. Sum-scores for the outcome measures were only calculated for participants who answered all questions for that particular outcome measure.

**Table 2:** Description of outcome measures and predictors

**Outcome measures**	
*Comprehension of information (5 questions) (composite: number of correct responses, range 0-5)*	
1.	Which hospital has procedures to prevent adverse effects of thrombosis?
2.	What is the performance of hospital A concerning the number of performed hip- or knee replacements among adults in a year?
3.	Which hospital is according to you the best when it comes to the conduct of doctors?
4.	Which hospital is the worst when it comes to procedures to prevent adverse effects of thrombosis?
5.	What is the performance of hospital B concerning the conduct of doctors?
Perceived usefulness of information (7 questions) (item: range 1-4*) (composite: range 7-28)	
1.	I think this information is handy
2.	I think this information is nice to look at
3.	I think this information is useful
4.	I think this information is not interesting (R)
5.	I think this information is important
6.	This information does not mean a lot to me (R)
7.	I would like to use this information if I had to make a hospital choice
*Hospital choice (1 question) (correct response; 0=wrong; 1=correct)*	
1.	Which hospital would you choose if you needed a hip- or knee surgery?
*Ease of hospital choice (1 question) (range -3 (very difficult) - 3 (very easy))*	
1.	How difficult was it for you to make a choice between the hospitals?
**Predictors**	
*Literacy (gap text; 5 missing words) (composite: number of correct responses, range 0-5)*	
*Numeracy (3 questions) (composite: number of correct responses, range 0-2^)*	
1.	Imagine that we flip a coin 1.000 times. What is your best guess about how many times the coin would come up heads in 1.000 flips? …times out of 1000
2.	In the ‘state lottery’, the chance of winning a €10 is 1%. What is your best guess about how many people would win a €10 prize if 1000 people each buy a single ticket? …person(s) out of 1.000
3.	In ‘the sponsor bingo lottery’, the chance of winning a car is 1 in 1.000. What percent of tickets of ‘the sponsor bingo lottery’ win a car? ….%^
*Active choice behaviour (search & selection behaviour scale) (6 questions) (item: range 1-4#) (composite: range 6-24)*	
1.	It doesn’t matter too much to me where and by whom I am treated.
2.	I don’t want to invest too much time and energy in the choice process.
3.	If I need care, I usually go the therapist/care facility to which my GP or specialist has referred me.
4.	If I need care, I usually investigate thoroughly how, where and from whom I will receive the best treatment (R).
5.	I have experience with the health care system and therefore know which therapist or care facility is best for me (R).
6.	I think it’s important to weigh possible treatments, therapists and care facilities against each other properly (R).

* These items use a four-point Likert-type scale. 1 = completely disagree 2 = disagree 3 = agree 4 = completely agree.

(R) Reversed item.

^ During the data collection period, an error in the system was discovered. Participants could only enter integers. Consequently, the answers on the third numeracy question (correct response 0.1) were not taken into account in the composite.

# These items use a four-point Likert-type scale. 1 = completely agree 2 = agree 3 = disagree 4 = completely disagree.

### Predictors

The predictor variables are also displayed in Table 2. We derived a measure for literacy from a language test of a university language centre in Belgium [32]. The test consisted of a gap text in which five words were missing. Participants had to choose out of five options the word that fitted in the text. Three items about numeracy were derived from a study of Schwarz and colleagues [33] and translated into Dutch. A composite (sum score of the number of correct responses) was developed for both literacy and numeracy to test these skills of participants.

To measure active choice behaviour, we used the search and selection behaviour scale tested and validated by Groenewoud [30]. The scale showed moderate reliability (α = .69). After recoding contra-indicative items, the scores on the six items were added up and a higher score represented a more extensive search and selection behaviour in the care process, that is: more active choice behaviour. Sum-scores of the predictors were only calculated for participants who answered all questions for that specific predictor. Finally, we added questions about age (seven categories), level of education (eight categories) and gender in the questionnaire for patients who enrolled themselves.

### Analysis

Data were analysed using STATA version 11.0. To check for differences in consumer characteristics (age, gender, education) and skills (literacy, numeracy, active choice behaviour) between the four subgroups, we performed a chi-square test (for gender) and Kruskall Wallis tests. Differences between the subgroups in the outcome measures were tested with Kruskall Wallis tests and a chi-square test (for correct hospital choice).

We conducted hierarchical multiple linear regression analyses to investigate the relative contribution of predictors to the comprehension of information, ease of making a choice and the usefulness of information. Three regression models were tested; the first model contained demographic characteristics, the second model contained demographic characteristics plus skills (literacy, numeracy and active choice behaviour), and the third model contained all these variables plus the amount of information. As the outcome measures and most predictors (except active choice behaviour) were not normally distributed and not measured at interval level, robust regression estimates (standard errors that do not assume normality) were used. We did not test regression models for participants’ hospital choice, due to the skewed distribution of this outcome measure. Alternatively, spearman’s rho correlations (spearman’s rho) were calculated to investigate the individual relationship between the predictors and this outcome measure. Results were considered statistically significant at the 5% level (p < 0.05).

## Results

### Respondents

In total, 414 people participated (response rate of 47%; Table 3). Of the respondents, 51 (12%) were patients and 363 (88%) were members of the Insurants Panel. The majority of the respondents was aged between 55 and 74 (71%) and was male (61%). A large part of the respondents had graduated from an advanced second level of education (35%) or had graduated from a lower second/vocational level qualification (19%).

**Table 3:** Demographic characteristics, skills and active choice behaviour of respondents

**Characteristics**	**Patients (N = 51)**	**Insurants Panel (N = 363)**	**Group 1 (N = 96)**	**Group 2 (N = 115)**	**Group 3 (N = 100)**	**Group 4 (N = 103)**	**Total (N = 413)**
Age (N, %)							
18- 24 years	0	0	0	0	0	0	0
25-34 years	1 (2.0)	13 (3.6)	1 (1.0)	4( 3.5)	6 (6.0)	3(2.9)	14 (3.4)
35-44 years	2 (4.0)	19 (5.2)	4(4.2)	8(7.0)	0	9(8.8)	21 (5.1)
45- 54 years	9 (18.0)	40 (11.0)	15(15.6)	13(11.3)	11( 11.0)	10(9.8)	49 (11.9)
55-64 years	19(38.0)	124 (34.2)	25 (26.0)	38(33.0)	40(40.0)	40(39.2)	143 (34.6)
65-74 years	14(28.0)	136 (37.5)	44(45.8)	41(35.7)	33 (33.0)	32(31.4)	150 (36.3)
75 +	5(10.0)	31 (8.5)	7(7.3)	11(9.6)	10(10.0)	8(7.8)	36 (8.7)
Gender (N, %)							
Male	17(34.0)	234 (64.5)	64 (66.7)	66( 57.4)	61(61.0)	60(58.8)	251 (60.8)
Female	33(66.0)	129 (35.5)	32 (33.3)	49(42.6)	39 (39.0)	42(41.2)	162 (39.2)
Highest level of education (N, %)							
No education	0	1 (0.3)	0	0	1 (1.0)	0	1 (0.2)
Primary school	1(2.0)	2 (0.6)	1(1.0)	1(0.9)	1(1.0)	0	3 (0.7)
Lower/preparatory vocational qualification	0	45 (12.4)	10(10.4)	12(10.4)	12 (12.0)	11(10.9)	45 (10.9)
Lower second/vocational level qualification	9(18.4)	70 (19.3)	16(16.7)	25(21.7)	20(20.0)	18 (17.8)	79 (19.2)
Intermediate second level general qual.	7(14.3)	50(13.8)	13(13.5)	14(12.2)	19(19.0)	11(10.9)	57 (13.8)
Intermediate vocational education	5(10.2)	38(10.5)	11(11.5)	14(12.2)	6 (6.0)	12(11.9)	43 (10.5)
Advanced second level education	17(34.7)	127(35.0)	36(37.5)	41(35.7)	31(31.0)	36(35.6)	144 (35.0)
Academic /higher vocational education	10(20.4)	30(8.3)	9(9.4)	8 (7.0)	10 (10.0)	13 (12.9)	40 (9.7)
Literacy (0-5) (Mean; SD)	4.9(0.3)	4.7(0.7)	4.8(0.7)	4.8 (0.7)	4.7(0.8)	4.9(0.6)	4.8 (0.7)
Numeracy (0-2) (Mean; SD)	1.6(0.6)	1.5(0.7)	1.4 (0.7)	1.5(0.7)	1.5 (0.7)	1.5 (0.6)	1.5 (0.7)
Active choice behaviour (6-24) (Mean; SD)	18.5(3.1)	17.1(2.8)	17.5 (2.6)	17.2 (2.8)	17.5(2.9)	16.8(3.2)	17.2 (2.9)

Group 1: 3 aspects shown, Group 2: 7 aspects shown, Group 3: 11 aspects shown, Group 4: 15 aspects shown.

No statistical significant differences were found between the four subgroups regarding consumers’ demographic characteristics and skills. The percentage of respondents who correctly answered all literacy items ranged from 83% (group 3) to 95% (group 4). The amount of respondents that answered all numeracy items correctly ranged from 55% (group 1) to 63% (group 4). In total, 59% of the participants had both numeracy questions correct and 88% had all literacy questions correct. As for the active choice behaviour, the average score was 17.2 (N = 413); given the scale range (6–24), this reflects a relatively high active choice behaviour. Group 4 had the lowest (16.8) and group 1 and 3 had the highest scores (17.5) on active choice behaviour (see Table 3).

### Differences in outcome measures between the four subgroups

Table 4 shows the scores of the four subgroups on the comprehension of information, the hospital choice, the ease of making this choice and the perceived usefulness of information. Differences between the subgroups were very small and not statistically significant.

**Table 4:** Mean and (SD) of outcome measures for the subgroups

	**Group 1 (N = 96)**	**Group 2 (N = 115)**	**Group 3 (N = 100)**	**Group 4 (N = 103)**	**Total (N = 414)**
Comprehension of information (range 0–5)	4.0(1.1)	3.8(1.2)	3.9(1.2)	4.1(1.1)	4.0 (1.1)
Correct hospital choice (0 = wrong; 1 = correct)	0.97(0.2)	0.97(0.2)	0.97(0.2)	0.99(0.1)	0.97 (0.2)
Ease of choice (−3 difficult until 3 easy)	2.0(1.4)	2.2(1.1)	1.9(1.6)	2.0(1.4)	2.0 (1.4)
Perceived usefulness of information (range 7–28)	21.5(4.0)	22.8(4.2)	22.6(3.8)	21.8(4.3)	22.2 (4.1)

Group 1: 3 aspects shown, Group 2: 7 aspects shown, Group 3: 11 aspects shown, Group 4: 15 aspects shown.

### The effect of the predictors on the outcome measures

The correlations between the predictors and choosing the best-scoring hospital were not statistically significant (ranging from −0.04 to 0.05). Table 5 displays the results of the hierarchical multiple linear regression analyses on data of 349 respondents.

**Table 5:** Hierarchical regression models with regression coefficients (Beta) predicting the outcome measures [N = 349]

	**Comprehension of information**			**Ease of hospital choice**			**Usefulness of information**		
	**Model 1**	**Model 2**	**Model 3**	**Model 1**	**Model 2**	**Model 3**	**Model 1**	**Model 2**	**Model 3**
**Predictors**									
Male (female = reference)	-.01	.04	.03	-.02	.02	.03	.01	.04	.05
Age	-.32**	-.28**	-.29**	-.16*	-.15*	-.15*	-.11	-.13*	-.12*
Education level	.29**	.18**	.18**	.16*	.09	.09	.10*	.06	.06
Literacy		.22**	.22**		.15*	.15*		.07	.08
Numeracy		.13*	.14*		.07	.08		.09	.09
Active choice behaviour		.04	.05		.13	.13		.24**	.23**
Amount of information									
3 aspects (reference)			-			-			-
7 aspects			-.12*			.11			.16*
11 aspects			-.08			-.05			.10
15 aspects			-.04			.01			.04
R^2^	0.19**	0.26**	0.27**	0.05**	0.09**	0.11**	0.02	0.09**	0.11**
Change in R^2^		0.07**	0.01		0.04*	0.02*		0.07**	0.02

* p < 0.05 ** p < 0.001.

For the outcome measure ‘correct hospital choice’ no regression models were estimated, due to the skew distribution of this outcome measure.

#### Comprehension of information

The third (full) regression model explained 27% of the variance in the comprehension of information. Age, education, literacy, numeracy and the amount of information were significant predictors of the comprehension of information. Age (ß = −0.29) was the most influential predictor and had a moderate effect; older participants had more difficulty comprehending the information that younger participants. The amount of information had a weak (ß = −0.12) but significant effect; participants provided with seven aspects had more difficulty comprehending the information than participants who saw three aspects. Inclusion of the amount of information in the third model did not result in a significant change in R-square (0.01).

#### Ease of hospital choice

The third (full) regression model explained 11% of the variance in the ease of making a hospital choice. Only age (ß = −0.15) and literacy (ß =0.15) had a significant but weak effect; younger and more literate participants considered it easier to choose a hospital compared to older and less literate participants. The effect of education in the first model disappeared in the third model. The increment in R-square in the final model (0.02) was significant, but the amount of information had no significant effect on the ease of choice.

#### Usefulness of information

The third (full) regression model explained 11% of the variance in the perceived usefulness of information. Age, active choice behaviour and the amount of information were significant predictors of the perceived usefulness of information. The active choice behaviour of participants (ß =0.23) was the most influential predictor and had a moderate effect. Younger and more active persons found the information more useful than older and less active persons. The inclusion of the amount of information did not result in a significant R-square change (0.02), but the amount of information did have a weak but significant effect (ß = 0.16). Participants who were shown seven items perceived the information as more useful than participants who saw three items.

## Discussion

To learn more about the influence of the amount of presented information on consumers’ use of comparative healthcare information, we studied this relation while taking into account important consumer characteristics and skills. We developed a test case of information about total hip/knee replacement surgery and focused on different indicators of effective information use, namely consumers’ comprehension of information, the choice of a hospital, ease of making a choice and perceived usefulness of the information.

This study showed that the amount of information slightly influenced the comprehension and perceived usefulness of comparative healthcare information. The effects of the amount of information, however, were not unambiguous and it remains unclear what the ideal amount of quality information to be presented would be. Furthermore, consumers’ demographic characteristics (especially age) and skills (especially literacy) were the most important factors affecting the comprehension of information and the ease of making a hospital choice. For the perceived usefulness of comparative information, active choice behaviour was the most influencing factor.

### Discussion of the results

The amount of presented quality aspects differentially influenced the different outcomes related to effective information use. Especially showing 7 compared to 3 quality aspects positively influenced the perceived usefulness of the information, but negatively influenced comprehension. There was no specific amount of information by which consumers comprehended the information the most, most often chose the best-scoring hospital, or perceived information as most useful. The demonstrated effects, however, do seem to correspond to what is known from the literature on interpreting and processing comparative healthcare quality information. Consumers are interested in the quality of care provided in hospitals [5] and the more aspects are presented, the more the information may meet their information needs [20]. However, in line with our expectations based on judgment and decision making psychology, presenting more quality aspects negatively influenced consumers’ comprehension of the information. Information comprehension was also strongly influenced by consumers’ educational level and literacy/numeracy skills. This could suggest that presenting more aspects particularly brings about problems for people with lacking skills.

The results of our study are not in line with previous findings of Peters and colleagues [2], who showed that the presentation of less information resulted in higher-quality hospital choices. In their study, people were shown five aspects (only cost and quality aspects) or nine aspects (cost, quality and non-quality aspects) in an ordered or unordered way about three hospitals. The different design and different aspects used in the two studies may explain the differences in findings. More specifically, the higher-quality hospital choice in their study was a hospital with the highest quality and the highest cost. This is a more difficult choice, as participants had to make a trade-off in their choice between quality and price. In our study, respondents did not need to make trade-offs, given that one hospital scored equally or better on all quality aspects compared to the other two hospitals. Considering the high levels of comprehension demonstrated in our study, we may well assume that participants who saw fifteen aspects did not feel anymore overwhelmed compared to participants who saw fewer aspects. This might explain why the effects of the amount of information were not very strong, and why the choice of the hospital and ease of this choice were not influenced by it.

Demographic characteristics and skills contributed most to the comprehension and perceived usefulness of information and the ease of making a hospital choice. Especially age seems to be an important aspect. Evidence is available for age-related changes in information processing and decision making [34]. When we become older, a decline in deliberative processes (our analytical, conscious mode of thinking; the opposite of our affective, spontaneous mode of thinking) will occur. As a result, older adults will be more likely to show some decision bias due to having difficulty with controlling attention and monitoring the accuracy of information in memory. Particularly in unfamiliar or less-meaningful situations, the decision making process of older adults is affected negatively. The aging-related decline in deliberative processes might explain the negative relation between age and the comprehension of information and ease of making a hospital choice in our study. On the other hand, older adults’ life experiences and associated knowledge can work as a compensating mechanism for age-related deliberative declines [34]. Being an experienced healthcare consumer, having a social network of other older and experienced healthcare consumers and relying on the expertise and information of healthcare professionals might help the aging population in making accurate healthcare choices [35,36]. This might explain why age contributed negatively to the perceived usefulness of comparative healthcare information in our study.

Previous research has also shown that health literacy and numeracy affect comprehension of information [29]. Furthermore, health literacy positively influences consumers’ confidence to make decisions as well as their ability to use comparative information in decisions [37]. Results about the role of active choice behaviour are more diverse. In our study, active choice behaviour only contributed to the perceived usefulness of information. Previous research demonstrated that patient activation (people’s knowledge, skill and confidence for managing their own health and healthcare [38]), is positively associated with seeking and using health information and making appropriate health choices [39,40]. Hibbard and colleagues [29] compared numeracy, health literacy and patient activation as predictors of the comprehension and use of comparative quality information. Though numeracy and literacy proved to be the strongest predictors, higher activation helped those low in literacy and numeracy compensate for their lower skill level and achieve higher levels of comprehension. A study of Nijman et al. [40], on the contrary, showed that patient activation was a stronger predictor for seeking and using health information than functional health literacy.

The amount of participants who correctly answered the numeracy items is worrisome; about 40% of the participants had difficulty with answering both questions correctly. Research of Schwarz and colleagues [33], of which the numeracy exercise was derived, also showed troubling results: 30% had zero correct answers and only 16% had three correct answers. If comparative healthcare information partly consists of numerical information, attention must be paid to the way this information is presented. Otherwise, this information will be only meaningful to consumers who have some facility with basic probability and numerical concepts [33].

Based on our results, we conclude that no ideal amount of quality aspects to be presented can be distilled, and that consumer characteristics and skills seem more important than the amount of quality aspects presented. We have to keep in mind, however, that in real life the decision making process can be more complex; consumers may have to compare more providers on even more quality aspects than shown in our study. Furthermore, comparative quality information might consist of more contradictory data about the quality of care (e.g. friendly doctors who, at the same time, have below average skills). We do not know if our participants took all the provided aspects into account or used only a subset of the information when deciding which hospital to choose [18,19]. More qualitative studies using existing quality data are needed to find explanations for the differential effects on different aspects of effective information use. For example, thinking aloud and eye-tracking methods could be used to analyse consumers’ information processing in detail when provided with different amounts of quality aspects.

Attempts to improve an effective use of comparative healthcare information by consumers may concentrate on both the presentation of the information and the skills of consumers. Given that the amount of information, consumer characteristics and skills together explained a relatively small amount of the variance in the outcome measures, other factors should also be considered when trying to increase the comprehension and use of comparative healthcare information. It may be worthwhile to consider presentation approaches that specifically improve effective information use for people with lower skill levels [2,24,41]. Providing a framework for the meaning of quality of care, using plain language, making quality measures easier to evaluate (e.g. by using well-tested symbols), presenting data in accordance with cognitive expectations of people (e.g. higher numbers reflect a better performance) and presenting frequencies (e.g. 2 out of 100) instead of percentages (2%) are possible presentation strategies to improve effective use of comparative healthcare information [2,42,43].

As consumers’ skills did have a moderate influence and both cognitive capacity and motivational aspects seemed to be important, we suggest that more attention is needed for improving consumers’ skills. According to Greene and colleagues (2005) [37], much work remains to be done to develop and test interventions that enhance the skills of consumers. Patient education programs, for instance, could focus on vulnerable groups known to have limited literacy and numeracy skills and aim to improve these skills. Notably, research has shown that patient activation is a changeable characteristic and in the United States interventions to improve patients’ involvement, knowledge and skills concerning their health and health care have been implemented successfully [44,45].

As a final point, although we favourite initiatives to improve the presentation of comparative healthcare information and consumers’ skills, we also have to keep in mind that not everyone is willing or mentally capable to search for and use comparative healthcare information and make choices on their own. Especially older people may have difficulties in fulfilling the role of active healthcare consumers. Employing a tailored approach, like social support of a healthcare professional or family member in the decision making process, may be more effective for these more passive or dependent consumers.

### Strengths and limitations

This study has several strengths. First, the information that participants were shown was based on actual information that is also presented in real life to consumers. Secondly, patients who underwent or had to undergo a total hip or knee surgery, and therefore are faced with this kind of healthcare choices in real life, were involved in this study. Because of the inequity in size between the group of patients and the group of members of the Insurants Panel, no comparisons could be made between these groups.

This research also has some limitations. First of all, it was limited to comparisons of hospital quality concerning a total hip or knee surgery. However, we do believe that our findings can be generalized to other choices for elective care, since the same type of decision-making processes apply. Second, people were not faced with a real choice; it was a hypothetical situation. In real life, this choice can be harder when no provider is explicitly superior, when contradictory information is presented and trade-offs have to be made, when more providers have to be compared or when emotions and personal experiences are involved. Finally, hospital A was considered as the ‘correct’ hospital choice, that is: it performed equally or better on all quality aspects. Far out most of the participants indeed choose hospital A and, as a result, we could not investigate the effect of the predictors on the hospital choice. Given that we do not know which factor (or factors) determined the participants’ decision, it remains unclear why some participants choose hospital B or hospital C. From the perspective of the participants, hospital B or C could still be a good choice.

## Conclusions

In current society more and more emphasis is placed on patients acting as active consumers when it concerns their healthcare choices. Reducing the amount of information that is presented, will probably not automatically result in a more effective use of comparative healthcare information by consumers in healthcare choices. In this context, more attention is needed for improving skills of consumers to manage this kind of information.

## Competing interests

The authors declare that they have no competing interests.

## Authors’ contributions

NZ developed the questionnaire and performed the data collection and analysis. She interpreted the data and drafted the manuscript. MH contributed to the conception and design of the study and helped to draft the manuscript. OD designed the study and contributed to the draft versions of the manuscript. EB performed extended data analyses and contributed to the draft version of the manuscript. JJ and SW coordinated the data collection in the Insurants Panel, critically evaluated the questionnaire and contributed to the draft versions of the manuscript. JR contributed to the conception and design of the study and the draft versions of the manuscript. All authors read and approved the final manuscript.

## Pre-publication history

The pre-publication history for this paper can be accessed here:

http://www.biomedcentral.com/1472-6947/12/101/prepub
